# Influence on Adiposity and Atherogenic Lipaemia of Fatty Meals and Snacks in Daily Life

**DOI:** 10.1155/2017/1375342

**Published:** 2017-06-19

**Authors:** Antonio Laguna-Camacho

**Affiliations:** Medical Sciences Research Centre, Autonomous University of the State of Mexico, Toluca, MEX, Mexico

## Abstract

The present work reviewed the connections of changes in consumption of high-fat food with changes in adiposity and lipaemia in adults with overweight or obesity. Hyperlipaemia from higher fat meals and excessive adiposity contributes to atherogenic process. Low-fat diet interventions decrease body fat, lipaemia, and atherosclerosis markers. Inaccuracy of physical estimates of dietary fat intake remains, however, a limit to establishing causal connections. To fill this gap, tracking fat-rich eating episodes at short intervals quantifies the behavioural frequency suggested to measure (by regression of changes in real time) direct effects of this eating pattern on adiposity and atherogenic lipaemia. Such evidence will provide the basis for an approach focused on a sustained decrease in frequency of fatty meals or snacks to reduce obesity, hyperlipaemia, and atherosclerosis.

## 1. Introduction

Atherosclerosis is globally a leading cause of death [1]. Atherogenic conditions, including dyslipidaemia, are associated with excessive adiposity [2]. The high prevalence of overweight and obesity in industrialised nations posits a serious public health concern [3]. This epidemic of obesity involves widespread patterns of unhealthy eating and physical inactivity. Although this research field is highly documented (i.e., [4]), the influence of habitual eating and exercise patterns on the atherogenic process remains to be measured as it occurs.

The current work articulates research on ingestion of dietary fat as a substantial contributor to the levels of adiposity and lipaemia. First, the physiological events are integrated from high-fat food consumption at eating occasions to infiltration of circulating atherogenic lipoprotein particles into the arterial wall. Then, evidence is presented for changes in adiposity, lipaemia, and markers of atherosclerosis by means of interventions to reduce dietary fat intake. Finally, a missing step for establishing causal links is pointed out together with a proposed approach for filling this gap.

**Figure 1:**
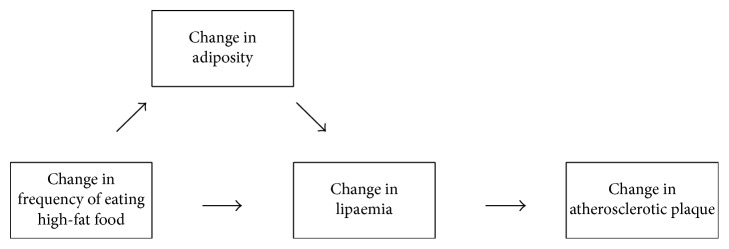
Behavioural model of influence of higher fat food consumption on adiposity and atherogenic lipaemia.

The present work considers also the behavioural field to move current research forward. The episodes of fatty meals or snacks are here addressed as a target behaviour pattern (Figure 1). The aim is to argue that episodes of high-fat intake one by one contribute to obesity, hyperlipaemia, and atherosclerosis. Therefore, a proposed implication is that individuals with overweight or obesity would benefit from consuming fewer fatty meals or snacks on a day to day basis than usual.

## 2. Dietary Fat Intake and Atherogenic Process

After an eating occasion, ingested fat is transported by lipoproteins for deposition as triglycerides within large intracellular lipid droplets in adipocytes without thermogenic cost. That is, lipids are more efficiently stored compared to carbohydrates or proteins. So a pattern of frequently consuming fatty meals or snacks would facilitate the expansion of body fatness. Accordingly, many studies have found association between high-fat diet and increased weight or body mass index [5, 6]. A focus on adiposity was recently proposed as this tissue is primary affected by hypertrophy of adipocytes, which contributes significantly to the inflammatory state found in obesity [7].

Lipolysis rate increases in response to excessive deposition of lipids in adipocytes [8–10]. Hence, hypertrophy of adipocytes contributes to higher release of free fatty acids to circulation. Also, people with overweight or obesity show an increase in plasma free fatty acids after a high-fat meal [11, 12]. The mechanisms of such postprandial abnormalities in human beings remain unclear. However, recent research shows that every episode of excessive fat intake causes adverse systemic effects by a peak of fatty acids in plasma [13, 14]. Thus, the more frequent a person consumes fatty foods, the more continuous also the physiological disturbances that contribute to atherosclerosis. In this case, the high flux of free fatty acids contributes directly to increase the synthesis in liver of lipoproteins that transport lipids in blood [15]. Thus, both high-fat meals and high adiposity cause hyperlipaemia in people with overweight or obesity. To provide an account of the process by which dyslipidaemia causes atherosclerosis, a biomechanical perspective is next considered.

There is an apolipoprotein B (apo B) molecule in the surface of each chylomicron (CM), very low-density lipoprotein (VLDL), intermediate-density lipoprotein (IDL), and low-density lipoprotein (LDL). Apo B has high molecular weight. Therefore, hyperlipaemia can elevate the blood volume or viscosity by higher number of apo B particles [16–19]. Vascular resistance compensates an increased volume of circulating blood [20]. The overstretching of the endothelium thickens the arterial wall and leads eventually to loss of compliance [21].

Endothelial failure contributes to slow blood flow as well as hematic stasis at sites of vascular resistance such as coronary arteries [22]. In state of hyperlipaemia, the blood stagnation at areas susceptible of lesion with diffuse thickening of the intima facilitates the infiltration of apo B containing lipoproteins into the arterial wall [23–25]. Lipoproteins are modified within the vascular endothelium [26]. Modified lipoproteins are a potent activator of immune cells [27]. Macrophages phagocyte modified lipoproteins in the vascular endothelium and release inflammatory cytokines such as interleukin 1, interleukin 6, and tumoral necrosis factor alpha (IL1, IL6, and TNF*α*) [28–30]. Macrophages release also chemokines to attract lymphocytes Th1 and Th2 that activate B cells that produce the antibodies immunoglobulin M (IgM) and G (IgG) against epitopes in the modified lipoproteins [30, 31]. Such immune response towards modified lipoproteins is a main factor of arterial inflammation [28, 32].

## 3. Effects of Prescribing to Decrease Dietary Fat Intake

Decreasing fat intake is a common strategy of dietetic interventions to reduce body fat and lipaemia [33–37]. Nondiabetic adults with overweight or obesity who take part in interventions based on low-fat or low-calorie diets, which usually involve strategies for decreasing dietary fat, show on average a reduction of 10% in body weight and 20% in fat mass as well as a decrease of 10% in apo B concentration [37–42]. There is also a rapid influence on apo B levels of dietetic changes. Decrease in apo B concentration of 10% was observed with a DASH diet within four weeks [43].

Prescribing dietary strategies to cut down on fat intake also decreases levels of modified lipoproteins and related immune biomarkers. For instance, reduction in body mass index by lifestyle intervention was associated with decrease in IL-6, protein-C reactive, normal T cell expressed and secreted, and other related markers of immune response and inflammation [44, 45]. People who lost weight in a long-term dietetic intervention showed also decreases in IL-1, IL-6, TNF *α*, and leukocytes [46].

One of the most studied modified lipoproteins has been the oxidised low-density lipoprotein (ox-LDL) [47]. Like other modified lipoproteins [48], a small fraction of the ox-LDL returns from the atherosclerotic lesion to the circulation [49]. So blood levels of ox-LDL could inform about the atherogenic process [50–55]. Interventions prescribing decrease in dietary fat intake have found parallel reduction in body weight or fat mass and in ox-LDL [56–58]. This is consistent with the mechanism that lowering fat intake and adiposity contributes to slow down the development of atherosclerosis. Indeed, there is reduction in the thickness of the atherosclerotic plaque over two years of maintained weight loss through low-fat diet [59].

## 4. Measurement Gap to Establish Causal Connections

Despite the well-documented positive association of changes in fat consumption with changes in fat mass and lipaemia, there is a significant bias in the core measure of dietary fat intake, the behavioural dose. Physical estimates of energy and nutrient content of diet are so inaccurate due to underreporting of food amounts that not using them in obesity research is suggested [60, 61]. Estimates are also limited by influence on intake of observing people's behaviour [62, 63]. Hence, researchers are unable to measure reliably enough in everyday life conditions if any prescribed dietary fat reduction causes the observed changes in adiposity, lipaemia, or inflammation biomarkers.

The effect of low-fat diets is taken for granted from outcome measures. However, even randomised controlled trials are not free of confounding effects from other unmeasured variables [64]. For instance, participants are liable to engage in unasked changes, which may include efforts of healthy eating, dieting, or exercising to reduce weight [65, 66]. Differences in baseline characteristics of participants can generate such behavioural responses that might account for the individual variability commonly observed in dietary interventions [67].

In any case, researchers rarely monitor the behaviour changes during dietary interventions. When change in dietary intake is monitored, it is usually at intervals of several months [68]. The effect of a decrease in dietary fat consumption could be achieved within a few weeks when such reduced rate of intake comes into balance with the lower energy expenditure from a reduced body weight [69, 70]. The outcomes of a behaviour change as they physiologically occur are still hardly considered. Identifying this prompt effect is needed for measuring the stepwise changes in adiposity and blood lipids caused by a particular pattern of eating such as eating less fat than usual at meals or between meals [71].

## 5. An Alternative Behavioural Measure to Narrow the Gap

Evidence from social psychology shows that people are highly accurate in their perception of what they normally do [72]. This psychological capacity helps people share efficiently information about their daily activities with other people. Giving a truthful account is cognitively less demanding than confabulating an event [73]. So researchers can collect reliable accounts of activities as they occur or elicit them without intrusion within the week period after their occurrence when the accuracy of recall is above 80–90% [74–77]. Such psychosocial measures of occurrence of everyday events include eating episodes. The count of a series of consecutive occurrences of a pattern of eating divided by the time of observation (i.e., number of episodes per week) gives a measure of its frequency, another dominant behavioural feature, instead of an estimate of energy/nutrient quantity or dose. Experimental designs that monitor episodes of any particular pattern of behaviour are well established in psychological science [78–80]. Tracking the episodes of an eating pattern informs about how often it is carried out over a time interval and how much a change in that frequency from an interval to another influences body fatness and related biomarkers. In the case of dietary fat consumption, the focus is on tracking episodes when people ate high-fat food at meals or between meals to measure the rate of its occurrence, which provides in turn a behaviour measure to regress changes in frequency of eating high-fat food onto changes in body weight, adiposity, blood lipids, and related immune or inflammation biomarkers. The slope of the regression gives a measurement of the effect on each of the outcomes per each unit of change in behaviour frequency.

In a small study to decrease unhealthy eating in adults with overweight or obesity, episodes of fatty meals and snacks were daily tracked during four weeks [81]. Participants reported a mean baseline of 12 high-fat meals or snacks a week, a frequency level that decreased throughout the intervention by almost half. Such sustained change in frequency of high-fat eating episodes correlated positively with change in fat mass and with change in ox-LDL. Also, the change in body fat correlated positively with change in ox-LDL.

This preliminary evidence tightens previous research showing that episodes of fatty meals and snacks over time are causally connected with change in adiposity and lipaemia as well as development of atherosclerotic plaque. Moreover, this shows that the effect of eating fatty meals and snacks on the arterial wall occurs within a few weeks.

Pursuing this research avenue would contribute to elucidating the causal links of behaviour patterns with obesity, dyslipidaemia, and atherosclerosis. This research line requires, however, substantial development. For instance, future studies could combine the present measure of frequency of an eating pattern alongside amounts of food eaten on each occasion estimated also with novel approaches that overcome limitations in accuracy of conventional dietary evaluations (i.e., [82]). A characterised pattern of eating would still correspond to a constant intake dose.

## 6. Concluding Remarks

Broad research shows consistently a positive association of fat intake with adiposity and apo B containing lipoproteins as well as with circulating levels of immune cells, cytokines, and modified lipoproteins. However, as gaps in research remain to accurately measure fat intake, there are still no measurements of causal connection between sustained intake of either more or less dietary fat and changes in anthropometry or physiological markers. The innovative use of psychosocial measures to collect records of the timings of the fatty meals and snacks as they are carried out by individuals in their everyday life would greatly benefit research and substantiate the evidence about the impact of dietary fat consumption on body fat, blood lipids, and biomarkers of atherosclerosis.

Research is beginning to accumulate showing that a bout of high-fat ingestion rapidly initiates metabolic impairments such as acute inflammation [13, 14]. Therefore, it is reasoned that frequent episodes of fat-rich food consumption contribute in real time to the low-grade inflammatory state associated with obesity that leads to cardiovascular complications.

Reducing the habitual number of episodes of unhealthy eating may contribute to minimising the physiological disturbances found in people with overweight or obesity. So monitoring fatty meals or snacks provides an approach based on behaviour frequency that readily generates evidence to inform objective recommendations against unhealthy eating. For instance, similar to the recommendation of exercising more than three times per week [83], a recommendation could be formulated of consuming higher fat food no more than six times a week, which is about half the frequency found in people with unhealthy body mass index [81].
